# Surgical Treatment of a Case of Ledderhose's Disease: A Safe Plantar Approach to Subtotal Fasciectomy

**DOI:** 10.1155/2015/509732

**Published:** 2015-12-10

**Authors:** Bruno Gonçalves Schröder e Souza, Gilberto Zaquine de Souza Júnior, Raíssa Mansilla Cabrera Rodrigues, Diogo Stelito Rezende Dias, Valdeci Manoel de Oliveira

**Affiliations:** ^1^Faculty of Medical Sciences and Health of Juiz de Fora (FCMS/JF - SUPREMA), BR 040, km 796, Salvaterra, 36045-410 Juiz de Fora, MG, Brazil; ^2^Hospital e Maternidade Therezinha de Jesus, Rua Doutor Dirceu de Andrade 33, São Mateus, 36025-140 Juiz de Fora, MG, Brazil

## Abstract

Plantar fibromatosis, Ledderhose's disease, or Morbus Ledderhose is an uncommon benign nodular hyperplasia of the plantar aponeurosis. The aim of this paper was to report the case of a 47-year-old male patient who had concomitant Dupuytren's disease and failed all conservative measures. He was treated surgically with prompt and complete relief of symptoms postoperatively, and he has had no recurrence at the 2-year follow-up. In this richly documented case, we discuss details of the surgical technique and anatomy, which was important for a successful outcome and preventing complications. The technique for subtotal fasciectomy is reviewed and the relevance of the adequate choice of skin incision to prevent painful scarring, skin necrosis, and difficulties with shoe wearing is highlighted.

## 1. Introduction

Plantar fibromatosis, Ledderhose's disease (LD), or Morbus Ledderhose is an uncommon benign condition of unknown etiology that was named after George Ledderhose who first described it in 1894. In LD, exacerbated proliferation of connective tissue produces thick collagen fibers, which forms slow growing irregular masses or nodules predominantly in the central band of the plantar aponeurosis [1, 2]. Such a pattern is also found in Dupuytren's disease (DD) of the hand, in which the palmar aponeurosis is affected, and a link between both diseases has been established. LD occurs in up to 14.85% of patients with DD [3]. Alcoholic hepatic dysfunction, diabetes mellitus, the use of anticonvulsants, and genetic factors may account for a higher risk of both diseases. Penile fibromatosis or Peyronie's disease has also been associated with this condition [2].

We report the case of a 49-year-old man who developed LP associated with Dupuytren's disease and discuss the options for treatment, the surgical technique, and details of pertinent anatomy [4].

## 2. Case Report

A 47-year-old Brazilian white man who worked in the metallurgical industry presented with a 2-year history of multiple slow growing nodules in the sole of the left foot, which was painful and prevented him from standing for a long time while wearing his work boots. He also complained of tender nodules and had a contracted band of the fourth finger of his right hand that prevented full extension; Dupuytren's disease was suspected. He presented with no other comorbidities, allergies, or medication use. He had a negative family history and denied alcohol abuse or diabetes mellitus.

**Figure 1:**
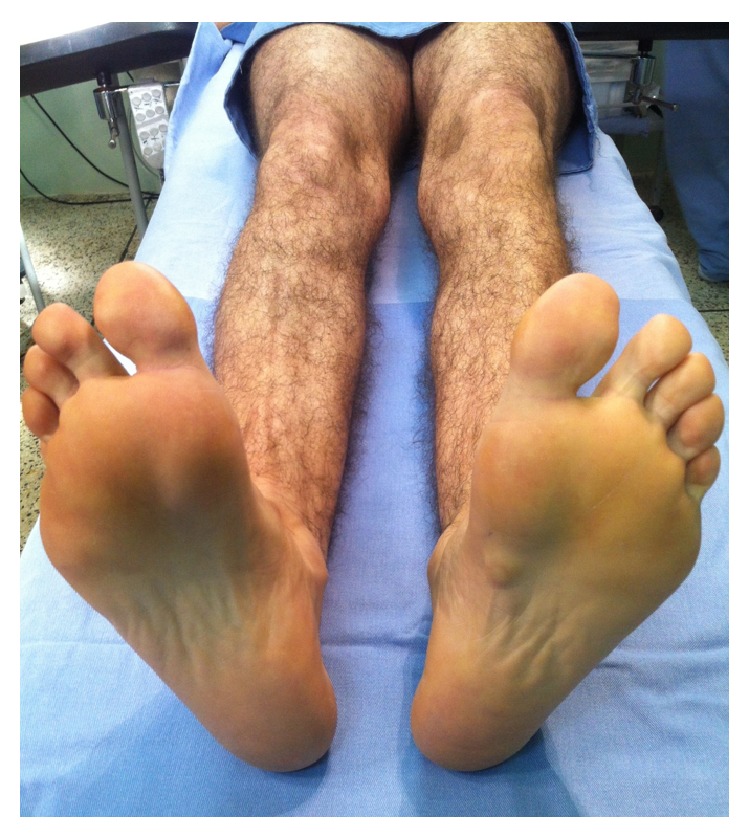
Nodules in the plantar aponeurosis of the left foot.

On examination, the feet were plantigrade and no deformities could be seen in the standing position. There was no noticeable limp; however, he reported claudication when the pain worsened. Examination of the left foot's sole showed three plantar nodules in the plantar fascia (Figure 1). On palpation, the nodules were painful and firm. They were located within the fascia, and they partially adhered to the skin. There were no flogistic signs. The neurovascular status was normal.

Foot radiographs were requested, which showed no calcifications or other specific alterations. Ultrasonography showed thickened fascia in the presence of nodules.

LD was diagnosed and conservative treatment was provided initially using a nonsteroid anti-inflammatory drug, custom-made silicon insole, home stretching exercises, cryotherapy, physical therapy, and counseling for proper footwear use. There was no improvement, and his pain and discomfort at work persisted after 6 months. Due to the continuous symptoms, surgical management was indicated. The patient was informed about our scientific interest in his case; he agreed to the proposed treatment and provided consent to publish this report. Subtotal plantar fasciectomy was performed.

**Figure 2:**
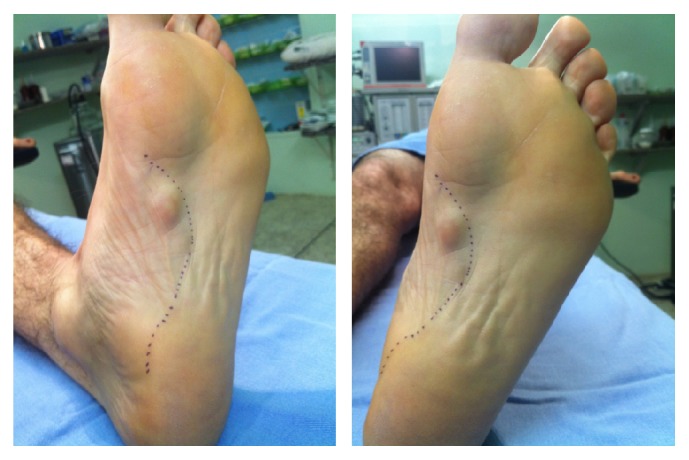
The planned S-curved shaped skin incision on the lateral aspect of the longitudinal plantar arch.

**Figure 3:**
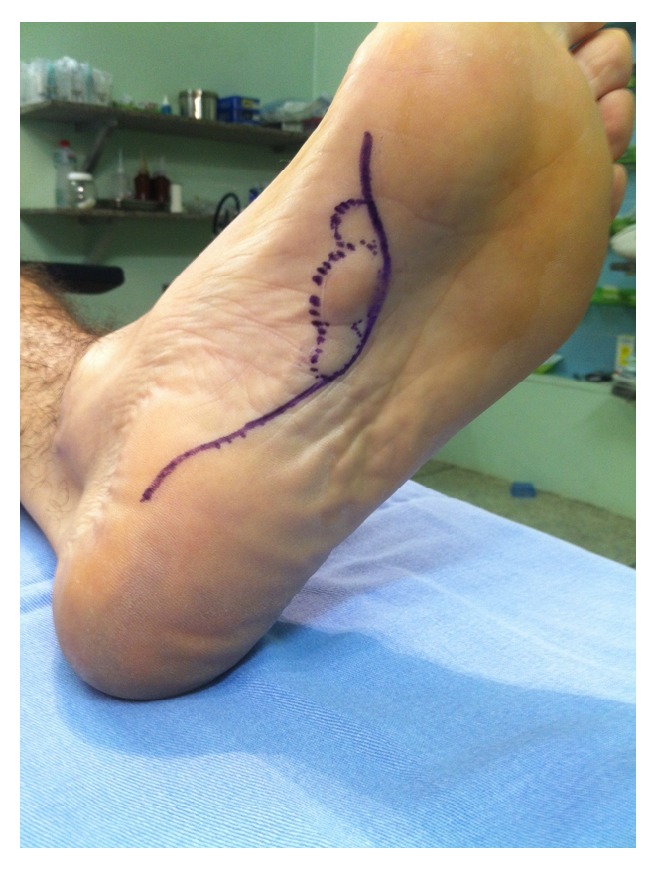
Detailed view of the three palpable nodules.

**Figure 4:**
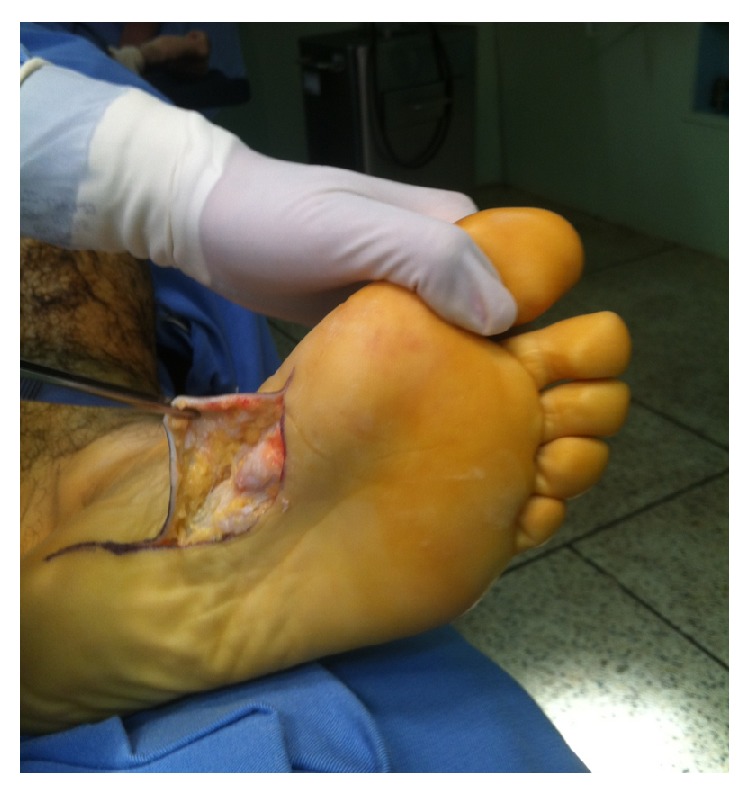
Dissection of the skin from the plantar fascia. Special care was taken to nourish the dermis.

**Figure 5:**
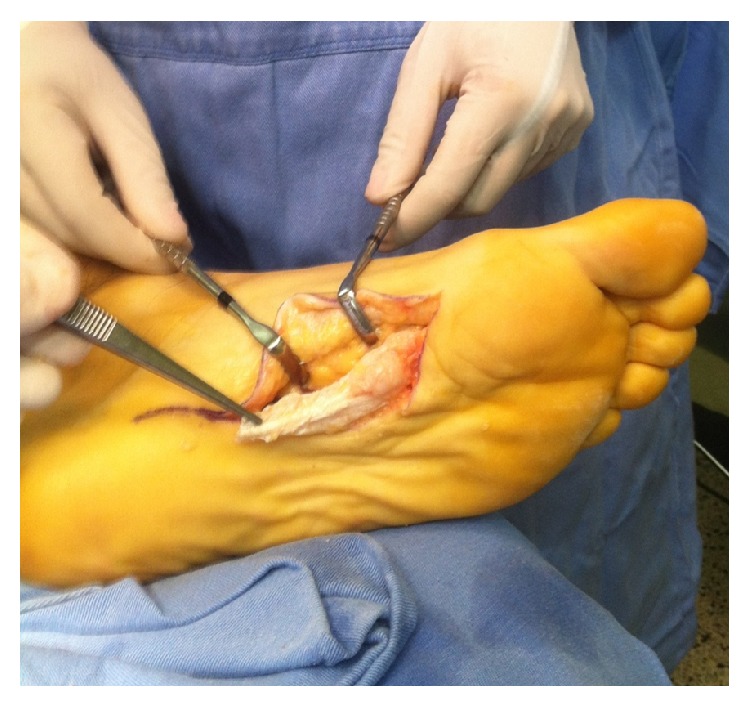
After isolating the plantar fascia, its proximal portion was transversely divided.

**Figure 6:**
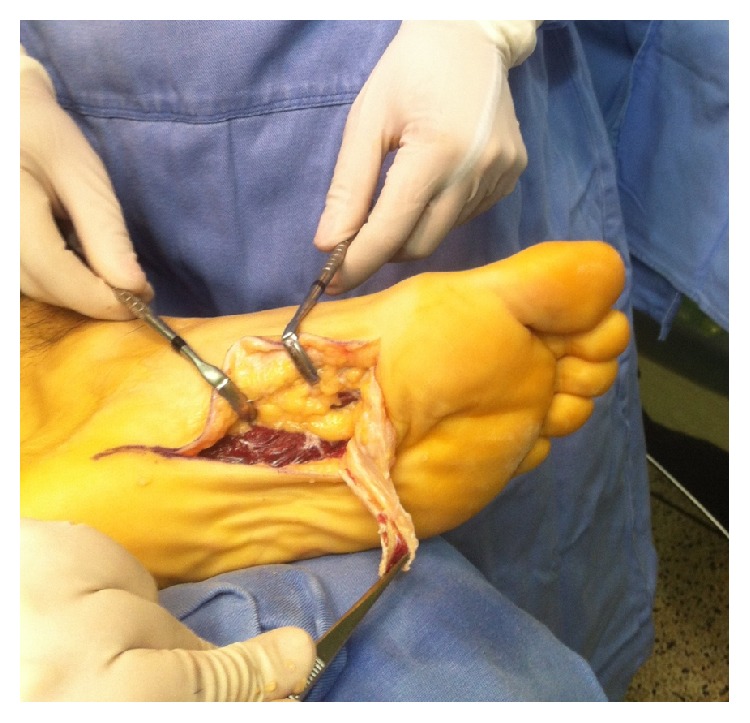
By reflecting the fascia distally, it was possible to protect the deep neurovascular structures.

**Figure 7:**
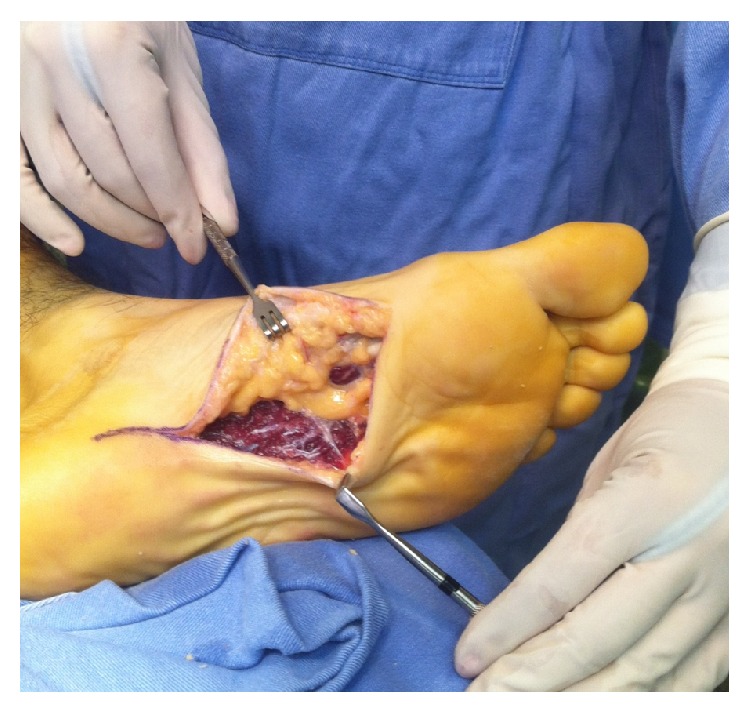
The final step after removing the plantar fascia.

**Figure 8:**
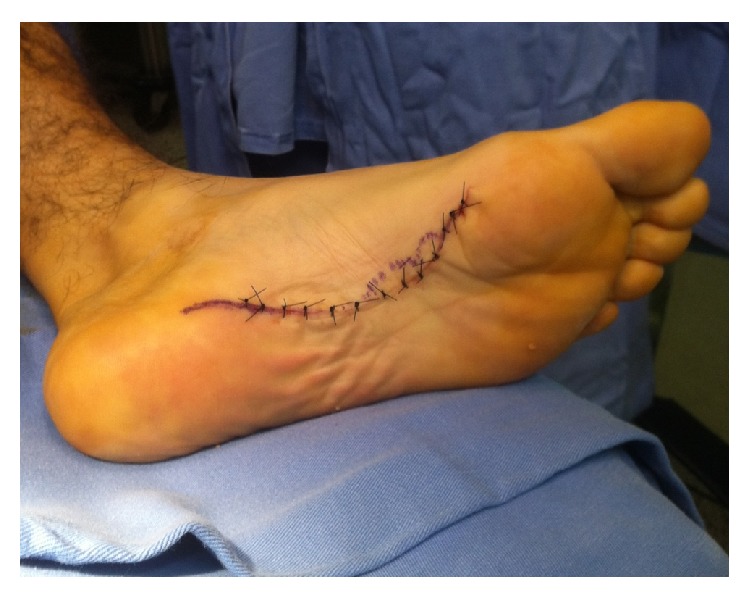
The sutured wound.

**Figure 9:**
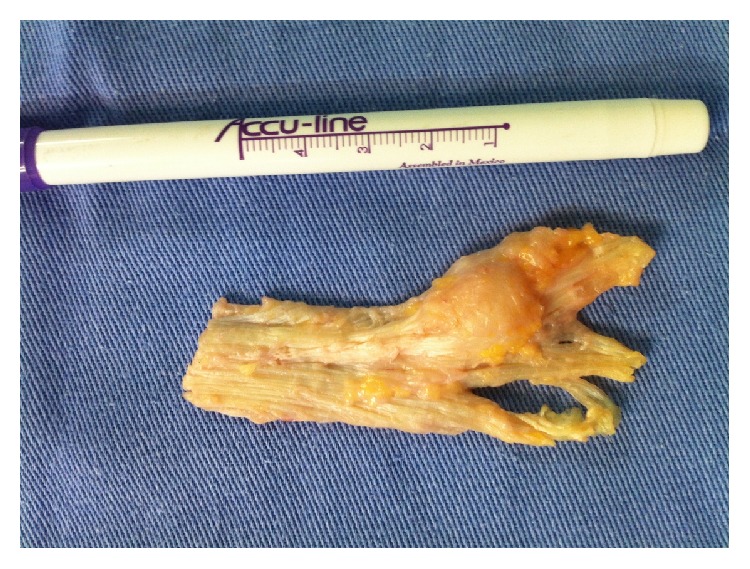
Macroscopic view of the resected tissue.

**Figure 10:**
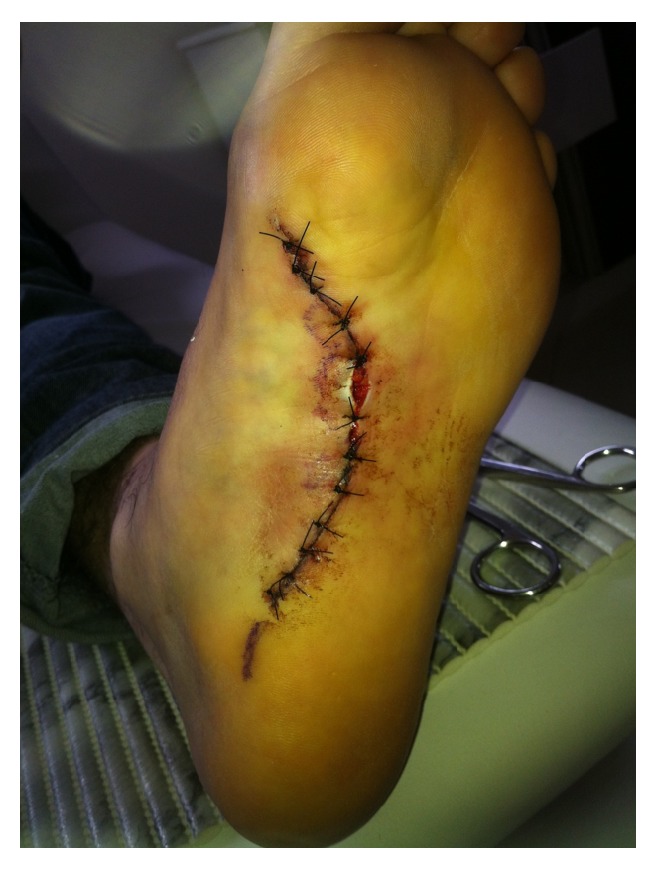
Good healing at 3-week follow-up, with only mild epidermolysis in the center of the wound.

The nodules were marked on the skin, and the planned surgical incision was drawn to include the nodules with respect to the local anatomy and physiology (Figures 2 and 3). An S-curved shaped incision was made just adjacent to the weight bearing surface (Figure 4). At this point, the underlying fascia adhered to the subcutaneous tissue, and careful dissection was performed to avoid undermining the skin and injuring the vascularity of the dermis. After the plane between the subcutaneous tissue and fascia was prepared, the plantar fascia containing the nodules was isolated (Figure 5), and it was transversely sectioned approximately 1 cm from its calcaneal origin to protect the underlying plantar nerves. Dissection was then performed distally, dissecting the plantar fascia from the underlying muscles and connective tissue where the plantar nerves could be visualized (Figure 6). Distally, the common plantar digital nerves were isolated and protected while emerging perpendicularly to the digit's fascia extensions. Next, those extensions were divided, and the excised tissue was sent to the pathologist (Figure 7). Devitalized and redundant skin was then removed (Figure 8). The skin was sutured under no tension (Figure 9). The dressings remained for 48 hours, and then they were changed daily. No weight bearing was allowed for 3 weeks, until the wound was reevaluated. There was no discharge, no inflammation, and only mild epidermolysis in the center of the wound (Figure 10). The sutures were removed without problems.

Histological examination of the excised tissue showed fibroblastic proliferation, with uniform and elongated nuclei but without mitotic figures, free margins, and cellular atypia.

**Figure 11:**
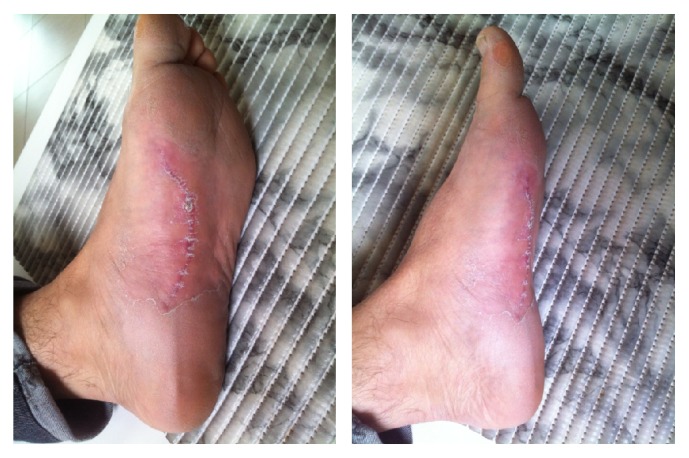
Normotrophic scar at 2-month follow-up.

After 4 weeks, there were signs of good healing, and full weight bearing using comfortable shoes was allowed. Two months later, the patient returned with eutrophic scar and no signs of retraction, nodules, or pain (Figure 11). He was not limping, and he was discharged to return to work with the use of a new custom-made silicone insole with arch support.

**Figure 12:**
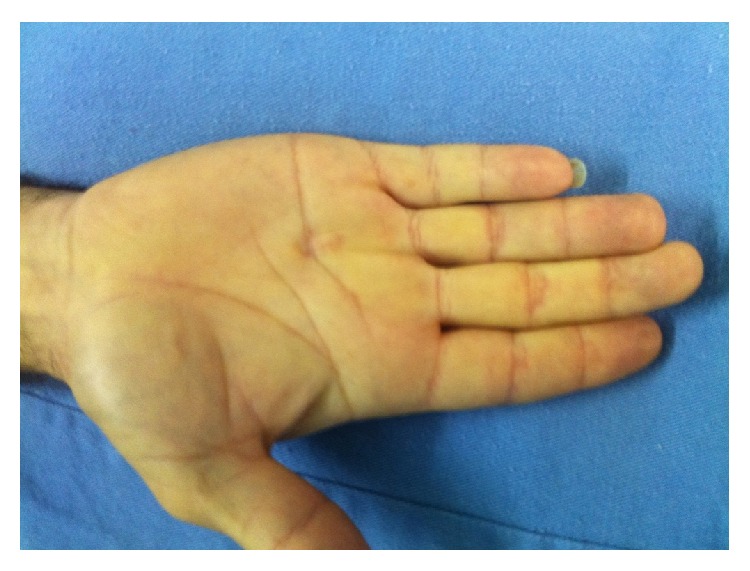
Dupuytren's disease involving the fourth ray of the right hand. The patient had good mobility after percutaneous fasciotomy.

After 3 months, a percutaneous needle fasciotomy [5] of his right hand was performed, with complete improvement of DD symptoms (Figure 12). At the 2-year follow-up, there was no sign of recurrence of LD, and he reported satisfaction and was able to work without any complaints. DD has not progressed, and no further symptoms or deformities have been observed thus far.

## 3. Discussion

LD refers to rare, benign hyperplasia of connective tissue of the plantar fascia. It is associated with conjunctival neoformation in the palmar fascia (i.e., DD) in 5–10% of cases and/or tunica albuginea of the penis (i.e., Peyronie's disease) in 1–3% of cases, of which LD is less frequent. Although there are some reported cases of genetic predisposition to LD, currently, no study has been able to prove this association. The condition can affect people of any age; however, it is more common in middle-aged patients or elderly men [2, 6], similar to the case presented herein. The diagnosis is essentially clinical, but ultrasound and magnetic resonance imaging can be obtained to confirm the diagnosis [7].

The disease presents as slow growing nodules usually found in the central and medial portions of one plantar fascia, although it may occur in both plantar fascias [8]. As with DD, three stages are described for LD: (1) proliferative with increased activity of fibroblasts; (2) involution with typical nodule formation; and (3) waste with a reduction of fibroblast activity, collagen maturation, and late tissue contractures.

Patients with LD may or may not present with clinical symptoms [6]. When positive, the symptoms usually include pain and difficulties adapting to shoes. The pain can be moderate to strong, and it can gradually worsen, especially in the standing position, due to compression exerted by fibrosis formation on the foot structures [1, 9].

Treatment options should be thoroughly evaluated, and conservative treatment is initially adopted to reduce the progression of symptoms. Nonsurgical treatment options include intralesional cortisone or collagenase injections [10], stretching, orthotics, anti-inflammatory drugs, physical therapy, shockwave therapy [11], and radiotherapy [12].

Surgical treatment should be indicated when other conservative measures have failed to provide symptom relief, which is rare [2, 9].  Table 1 presents all cases reported in the literature that have been surgically treated. The standard procedure consists of subtotal fasciectomy, as it represents a less invasive option with good results. Nevertheless, total fasciectomy may be indicated for some patients with adjuvant radiotherapy treatment seeking to reduce the recurrence of lesions. No specific risk factors have been reported for the recurrence of LD. However, risks factors for the recurrence of DD have been reported and include disease onset in those younger than 50 years, a bilateral disease, LD, first ray involvement, multiple ray involvement (>2 digits), ectopic fibromatosis, a family history, and male sex. No correlation was observed between self-reported disease recurrence and those with diabetes, frozen shoulder syndrome, or epilepsy [13].

Complications of surgical treatment include skin necrosis and dehiscence, infection, retracted and painful scarring, and difficulties with shoe wearing. Additionally, numbness or painful neuromas can occur when branches of the plantar nerves are injured during surgery. To avoid such events, the anatomy and physiology of the plantar region of the foot should be clearly understood to reduce the chance of permanent damage.

**Figure 13:**
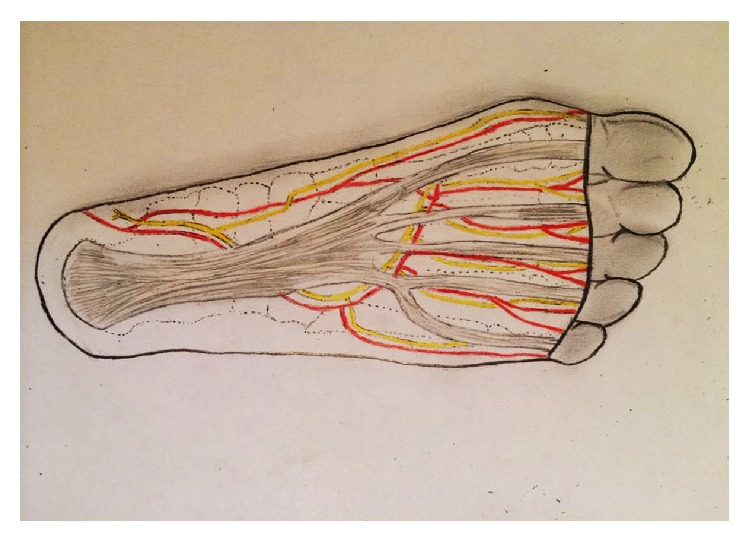
Schematic drawing of the anatomic relationship of the plantar fascia and the neurovascular structure of the foot's sole.

Arteries in the plantar aspect of the foot consist of terminal branches of the posterior tibial artery. Although the medial plantar artery supplies blood to the medial plantar skin via its superficial branches and occasionally forms the superficial plantar arc, the lateral plantar artery bends medially to form the deep plantar arch, making a variety of perforating branches responsible for providing vascular supply to the remaining skin [14, 15]. It is important to protect the arterioles that nourish the dermis, leaving the fascia perpendicular to the skin and curved in a centrifugal way (i.e., from the midline of the foot toward its medial and lateral aspects) [1] (Figure 13). This anatomical feature explains why longitudinal or zigzag incisions medial to the midline of the foot can increase the risk of ischemia and necrosis of the skin on the medial side of the wound. Besides, longitudinal incisions along the medial plantar arch that are distant to this anatomical peculiarity can produce hypertrophic and painful scars because they transverse the tension lines of the skin (i.e., Langerhans' lines). Additionally, incisions crossing the loading area (i.e., the lateral aspect of the foot) are not recommended because they usually generate discomfort or pain while walking. Therefore, the incision we used seemed to be less prone to such complications, and we advocate its use.

**Figure 14:**
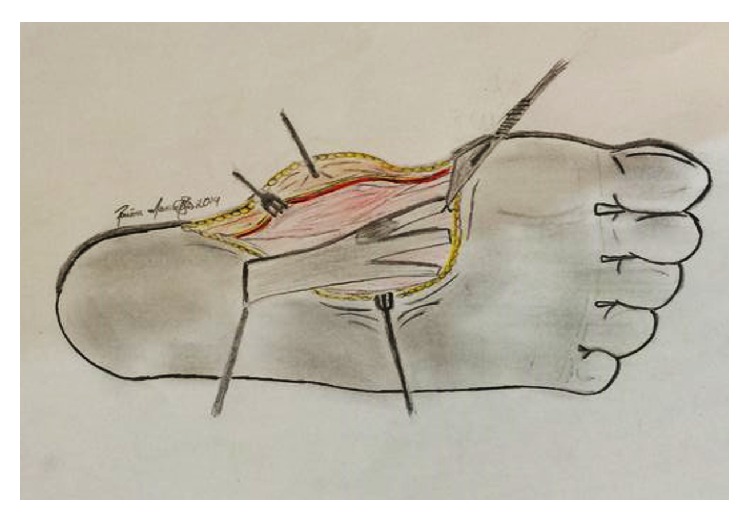
Schematic drawing of the division of the fascial extensions to the toes. Care should be taken to protect the emerging digital nerves as they cross these extensions.

Regarding the nerves, we emphasized the importance of the medial plantar nerve and the lateral end branches of the tibial nerve that originate in the common plantar nerves. These branches become superficial between the digital projections of the plantar aponeurosis and induce sensitive innervation in the toes. As they cross the commissures of the plantar fascia, dissection at these areas should be performed carefully to avoid permanent paresthesia or anesthesia [14] (Figure 14). Dissecting the fascia from its deeper portion after releasing its proximal origin may help to identify, dissect, and protect the plantar nerves and its branches (Figures 6 and 14).

## 4. Conclusions

Although rare, LD can be a disabling disease to symptomatic carriers. The present case described surgical management of this condition and highlighted the importance of several anatomical and physiological details that are paramount for the success of this procedure.

## Figures and Tables

**Table 1:** List of published cases of Ledderhose's disease.

Authors	Year	Title	Number of cases	Number of surgical cases	Journal
Kan and Hovius	2012	Long-Term Follow-Up of Flaps for Extensive Dupuytren's and Ledderhose Disease in One Family	2	2	J Plast Reconstr Aesthet Surg

Nikolić et al.	2011	Plantar Fibromatosis and Dupuytren's Contracture in an Adolescent	1	0	Vojnosanit Pregl

Koudela Jr et al.	2010	Plantar Fibromatosis (Ledderhose's Disease)	1	1	Acta Chir Orthop Traumatol Cech

van der Veer et al.	2008	Recurrence of Plantar Fibromatosis after Plantar Fasciectomy: Single-Center Long-Term Results	27	27	Plast Reconstr Surg

Fetsch et al.	2005	Palmar-Plantar Fibromatosis in Children and Preadolescents: a Clinicopathologic Study of 56 Cases with Newly Recognized Demographics and Extended Follow-Up Information	56	56	Am J Surg Pathol

de Almeida Jr et al.	2001	Plantar Fibromatosis with Marked Cutaneous Involvement	1	1	Hautarzt

Jacob and Kumm	2000	Benign Anteromedial Plantar Nodules of Childhood: A Distinct Form of Plantar Fibromatosis	1	0	Pediatr Dermatol

Sammarco and Mangone	2000	Classification and Treatment of Plantar Fibromatosis	18	18	Foot Ankle Int

Godette et al.	1997	Plantar Fibromatosis of the Heel in Children: A Report of 14 Cases	14	6	J Pediatr Orthop

Aluisio et al.	1996	Plantar Fibromatosis: Treatment of Primary and Recurrent Lesions and Factors Associated with Recurrence	30	30	Foot Ankle Int

Parnitzke et al.	1991	“Ledderhose” Disease. Plantar Fibromatosis–Clinical Aspects	7	7	Zentralbl Chir

Pentland and Anderson	1985	Plantar Fibromatosis Responds to Intralesional Steroids	1	0	J Am Acad Dermatol

Bottinelli	1982	Ledderhose Disease (Case Considerations)	30	30	Chir Ital

Westerkamp	1978	A Case History of Recurrent Plantar Fibromatosis (Dupuytren's Contracture)	1	1	J Foot Surg

Total			190	179	
